# Influence of Using Perforated Plastic Flooring Beneath the Waterline on Growth Performance, Litter Quality, and Footpad Health of Broiler Chickens: A Field Study

**DOI:** 10.3390/ani12141749

**Published:** 2022-07-07

**Authors:** Sylvan-Justin Sonnabend, Fabian Spieß, Bernd Reckels, Marwa F. E. Ahmed, Amr Abd El-Wahab, Christian Sürie, Jan Berend Lingens, Christian Visscher

**Affiliations:** 1 Institute for Animal Nutrition, University of Veterinary Medicine Hannover, Foundation, Bischofsholer Damm 15, D-30173 Hannover, Germany; sylvan-justin.sonnabend@tiho-hannover.de (S.-J.S.); fabian.spiess@tiho-hannover.de (F.S.); bernd.reckels@tiho-hannover.de (B.R.); marwafawzy@mans.edu.eg (M.F.E.A.); amrwahab5@mans.edu.eg (A.A.E.-W.); christian.visscher@tiho-hannover.de (C.V.); 2 Department of Hygiene and Zoonoses, Faculty of Veterinary Medicine, Mansoura University, Mansoura 35516, Egypt; 3 Department of Nutrition and Nutritional Deficiency Diseases, Faculty of Veterinary Medicine, Mansoura University, Mansoura 35516, Egypt; 4 Farm for Education and Research Ruthe, University of Veterinary Medicine Hannover, Foundation, Schäferberg 1, D-31157 Sarstedt, Germany; christian.suerie@tiho-hannover.de

**Keywords:** broiler, slatted flooring, performance, footpad dermatitis

## Abstract

**Simple Summary:**

The standard housing method in Europe’s poultry farming is to keep broilers on a littered concrete floor. Therefore, wood shavings are used to catch moisture to prevent wet litter. In particular, the broilers frequently visit the wet area beneath the waterline, where the wet litter causes the main problem in footpad health and animal welfare. The current study tested the impact of slatted flooring beneath the waterline by dividing the barn into three areas (drinkers, feeders, comfort) in terms of growth performance, litter quality, and footpad health of the broilers. The quality of the litter was assessed on the basis of different parameters, for example, dry matter. No effect on growth performance between a littered floor and partially slatted flooring was noted. However, the litter parameters show a small positive impact on the litter quality, especially in the areas of drinkers and comfort, while using partially slatted flooring. Nevertheless, the slatted flooring had an impact on the footpad health of the broilers, with a lower FPD score at the end of the fattening period.

**Abstract:**

The aim of this study was to evaluate the effect of slatted flooring beneath waterlines in broiler barns on litter quality and, subsequently, footpad health. The hypothesis tested was that installing slatted flooring underneath waterlines helps to improve litter quality and thus reduces footpad diseases, enhancing animal welfare as a result. Five experimental runs with two groups were conducted. Each run was defined as one fattening period of 32 days and consisted of 15,000 broiler Ross 308 of both sexes. Every barn was divided into three areas (drinkers, feeders, and comfort area) for weekly sampling. No influence on growth performance was noted. The slatted flooring influenced the litter quality by preventing the litter in the experimental group (EG = 690 ± 167 g/kg DM) from becoming moisture until day 14 of the fattening period compared to the control group (CON = 636 ± 198 g/kg DM). The footpad health was also influenced by using slatted flooring, with lower camera-based footpad scores in the EG (8.80) compared to CON (22.0) at the slaughterhouse (*p* = 0.0258). Installing slatted flooring beneath the waterline reduced the moisture of the litter compared to the control barn in the first two weeks of age and showed a positive effect on the footpad health of the broilers at the end of fattening, which indicates an improvement in animal welfare.

## 1. Introduction

In today’s poultry farming, housing conditions and management significantly influence animal health and welfare [1]. In Germany’s commercial chicken farms, broilers are predominantly kept on a littered concrete floor [2]. The European Council decided that the “useable area” for chickens kept for meat production is a littered area accessible to chickens at any time [3]. The litter not only serves as a bedding material [4], but also should allow the animals to demonstrate the essential species-specific behavior of pecking, scratching, and taking a dust-bath, thus improving animal welfare [5,6]. Poor litter quality (wet litter) leads to footpad dermatitis (FPD) in poultry and consequently may affect animal welfare negatively [7,8,9,10,11,12]. The necrotic and ulcerative lesions of the footpads can be painful and cause stress [13] as well as provide an entry point for pathogens that can negatively affect overall health [14]. Reduced performance, such as low feed intake, can result in decreased final weight and poor feed conversion [8].

The moisture levels in commonly used bedding materials, such as wood shavings and sawdust [15], can also impair the health and the welfare of the animals when the moisture of the litter, through excreta, exceeds the evaporation of the water in litter [16]. Several intrinsic and extrinsic factors can affect litter quality, such as seasons, air humidity, watering design, excrement consistency, and stocking density [11,17,18,19,20]. Additionally, nutrient imbalances, such as unbalanced protein levels, minerals, or vitamins, can affect footpad health [21,22,23,24,25]. In particular, the management of the waterline can influence the FPD through exposure to wet litter [26]. Therefore, different litter materials, floor heaters [25,27], and plastic floor constructions [28] have been used to improve litter quality and thus prevent FPD [11,29,30]. Perforated plastic flooring is very durable, cost-effective, solid, easy to build, and clean [31]. However, plastic constructions may also have some negative influences, such as mechanical pressure on the footpads and decreased body weight [7,8,32]. Nonetheless, previous studies have addressed the concept of a perforated level by also analyzing the aspects of performance and footpad health [33,34,35].

Different systems trying to separate broilers from their excrements by perforated layers have been carried out in some areas of the world. The rearing of laying hens [36] as well as broiler producers in parts of South Africa and Russia [37] reflect examples of working systems. This fully perforated flooring system has always been used to reduce the contact between animals and excrement. However, a level system without litter could negatively affect animal species-specific behavior, which would interfere with animal welfare [38]. Therefore, the Welfare Quality Assessment protocol, which is based on four main principles, such as good feeding, good housing, good health, and appropriate behavior, shows that fully perforated flooring systems are no alternative to littered flooring systems to fulfill animal welfare [39].

The objective of this study is to install a partial slatted flooring beneath the watering line within a littered house, which is designed to prevent the broilers from coming into contact with wet litter and creating dry litter comfort zones within the house. The hypothesis of the study is that by implementing partially slatted flooring beneath waterlines, it is possible to create comfort areas with dry litter and consecutively reduce the severity of FPD. This should give the broilers better housing conditions without there being a negative influence on growth performance.

## 2. Materials and Methods

The broiler chickens in this study were raised under standardized husbandry conditions and subjected to a standard fattening procedure on the Farm for Education and Research Ruthe, University of Veterinary Medicine Hannover, Foundation, Sarstedt, Germany. Since no interventions were carried out on live animals, the study was not considered an animal experiment in accordance with the Animal Protection Act and thus did not require approval from the respective authority.

### 2.1. Animal Housing and Experimental Design

The study was performed in five consecutive runs with approximately 15,000 broilers (Ross 308) of both sexes per run. Each run was defined as one fattening period of 32 days. One-day-old chicks were obtained from a conventional hatchery (BWE-Brüterei Weser-Ems GmbH and Co. KG, Visbeck, Germany) and were divided into two groups per run: the control group (CON) and the experimental group (EG). Every run was carried out in two barns: 8000 animals in the CON group (29.5 m × 15.9 m = 469 m^2^ = 17.05 animals/m^2^) and 7000 animals in the EG group (35.6 m × 11.3 m = 402.3 m^2^ = 17.4 animals/m^2^). The control barn contained three feeding and four watering lines, which were symmetrically arranged. The experimental barn was symmetrically oriented with two feeding lines and three drinker lines. Both barns were littered once with conventional wood shavings (GOLDSPAN, Goldspan GmbH and Co. KG, Goldenstedt, Germany) at the beginning of each run. Within the fattening period, no litter was substituted. The climate in each barn was adapted to the broilers’ needs (Aviagen, 2019) [40]. The temperature and humidity were measured through sensors and a regulated climate computer (ViperTouch; Big Dutchman International GmbH, Vechta, Germany), which controlled the negative pressure ventilation system in both barns. In the first few days, the temperature was set to 35 °C and was reduced to 20 °C by the end of the experiment. Humidity increased from 30% on the first day of fattening to 67% by the age of slaughter. The light intensity was identical in both groups for the five runs. The dark period was between 21:00 h and 05:00 h (16 L:8 D). Both lights and shutters, which blocked external light, were automatically controlled using timers. The birds were vaccinated via drinking water at d 12 with Poulvac ND Hitcher B1 (Pfizer GmbH) against New Castle, at d 18 with AviPro Precise (Lohmann Animal Health GmbH & Co. KG Cuxhaven, Germany) against Gumboro and at d 20 with Nobilis IB Ma5 (MSD Animal Health GmbH) against infectious bronchitis virus strain Ma5.

**Figure 1:**
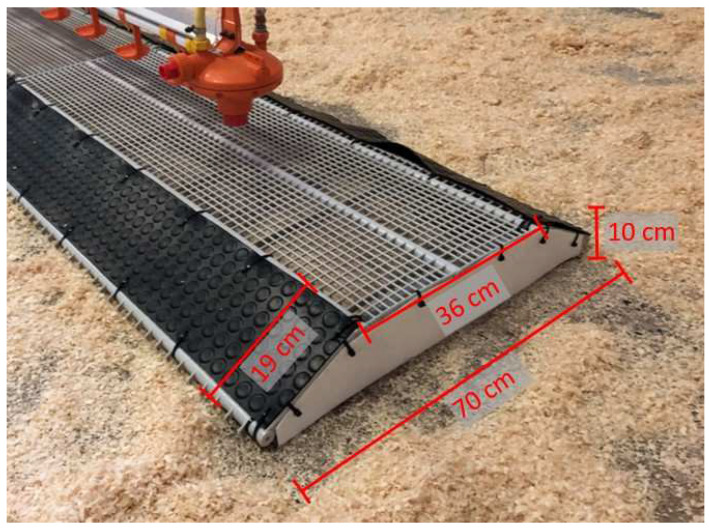
A trapezoid slatted floor.

The experimental barn was equipped with 80 slatted floors beneath the drinker lines. The slatted floors beneath a drinker line were combined to form a whole element covering about 30 m. Each level segment made of slatted flooring consisted of holes (15 × 10 mm) and bridges (plastic-covered steel; width 3.5 mm; Big Dutchman International GmbH, Vechta, Germany) shaped like a trapezoid (Figure 1).

### 2.2. Diets

Feed and water were available ad libitum for all groups. Both barns had the same batch of feed for each run. All birds in both trials were fed commercial pelleted diets throughout the rearing period (Table 1). The commercial starter diets were offered for the first week of life to all birds. From the beginning of the second week, the birds received a commercial grower I diet. From d 21 of life, the birds were fed a commercial grower II diet. Finally, the commercial finisher diet was offered from d 28 of life to all birds. Generally, the commercial diets fed to all birds during all runs were based on wheat grain, yellow corn, soybean meal, and rapeseed meal obtained from a local feed company (MEGA Tierernährung GmbH & Co. KG, Visbek-Rechterfeld, Germany). Zootechnical supplements such as 100 U endo 1.4-β-glucanase, 88 U endo 1.3(4)-beta-glucanase, 342 U endo 1.4-beta-xylanase and 1.000 U 6-phytase were added by the feed manufacturer.

### 2.3. Measurements

#### 2.3.1. Growth Performance

**Figure 2:**
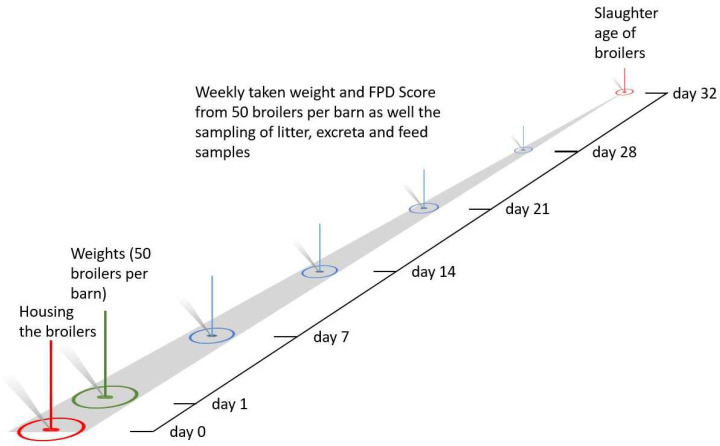
Sampling timeline for each barn in each run.

In each fattening run (Figure 2), every week, the average body weight (BW) of 50 randomly selected broilers per barn was manually recorded using hanging poultry scales (BAT 1, VEIT Electronics, Moravany, The Czech Republic). In addition, the number of delivered birds and the total weight of all birds in each barn run were determined at the slaughterhouse, and the average live weight was calculated after subtracting the rejected birds. For each run, the total feed and water consumption were noted to calculate the daily feed and water intake from day 0 to 33. Additionally, the water-feed ratio was calculated by dividing the daily water intake by the daily feed intake per barn. The mortality-corrected feed conversion ratio (FCR) was determined for each run, as shown by Dersjant-Li et al. [42]. The average daily gain (ADG) for days 0–33 was calculated by dividing the body weight by the days of each run.

#### 2.3.2. Excreta and Litter Quality and Foot Pad Scoring

During each run, fresh excreta samples with the least possible amount of visual contamination (*n* = 6) from each group were collected (with pooled amounts of about 50 g) weekly to measure DM content, nitrogen, calorific value, and starch. Additionally, litter samples (*n* = 92) were collected (on the same day as the excreta sampling).

**Figure 3:**
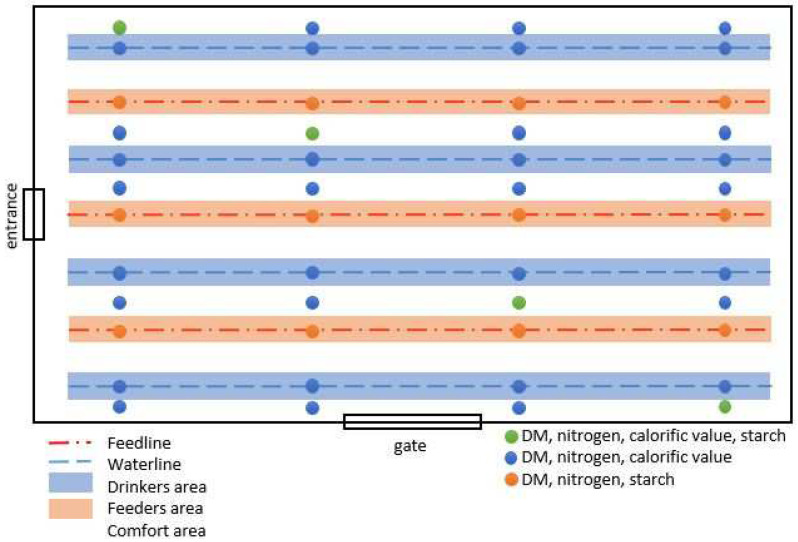
Shown is the sampling plan of the control barn. The blue (dashed) and red (dash-dotted) lines are the water and feeding lines. Therefore, the blue and red areas are the drinker and feeder areas. The white space is the comfort area. The green sample points were analyzed for DM, nitrogen, calorific values and starch, whereas the blue sample points were analyzed for DM, nitrogen and calorific values. The red sample points were analyzed for DM, nitrogen and starch.

**Figure 4:**
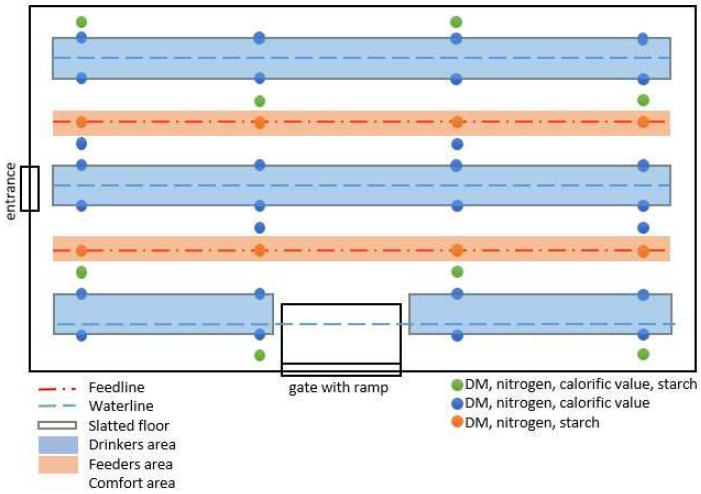
Shown is the sampling plan of the experimental barn. The blue (dashed) and red (dashed-dotted) lines are water and feeding lines. Therefore, the blue and red areas are the drinker and feeder areas. The white space is the comfort area. The green sample points were analyzed for DM, nitrogen, calorific value and starch, whereas the blue sample points were analyzed for DM, nitrogen and calorific values. The red sample points were analyzed for DM, nitrogen and starch.

The litter samples were taken according to the following sampling plans shown in Figure 3 and Figure 4. In order to guarantee comparability, each barn was divided into three areas (drinkers, feeders, and comfort area). The drinker area was located beneath the waterline. In CON, samples could be collected right underneath the waterline, whereas in EG, due to the slatted flooring, samples had to be collected from the side of the slatted flooring. Underneath the feeding line, samples were collected next to the feeding pods. The remaining space in the barn was defined as a comfort area. At each sample point, about 100 g of excrement-litter material was taken by hand right down to the depth of the barn floor. Depending on the sample point, all samples were analyzed for DM and nitrogen content. For each run, 72 samples were analyzed calorimetrically for calorific value (blue + green points) and/or 32 samples enzymatically for starch (orange + green points) (Figure 3 and Figure 4).

The foot pads (only the central plantar) of the birds (50 birds/group) were scored weekly by the same veterinarian on a scale from 0 to 7 in accordance with Mayne et al. [12]: score 0 = healthy skin, score 7 = more than 50% of foot pad area is necrotic. The scoring of each leg was done for each bird. Footpad scores were determined by calculating the average of the scores for both feet of each animal.

Moreover, at the end of the fattening period, camera-based footpad scoring (QS Fachgesellschaft Geflügel GmbH Germany) [43], with a scale of 0, 1, 2a, 2b, which is commonly used at the slaughterhouse, was performed. The final score depended on the severity of the footpad. Therefore, the percentage of each score (0; 1; 2a; 2b) was multiplied by a factor (0; 0.5; 1; 2) and added together. Accordingly, a high score indicated poor footpad health.

### 2.4. Analysis

The samples were analyzed according to the standard and official methods of the VDLUFA [44]. To determine the DM, the samples were weighed and freeze-dried for three days at −20 °C. Afterwards, the samples were weighed again and ground to determine the second DM. Starch was calculated enzymatically (Stärke UV-Test, Boehringer Mannheim/R-Biopharm, order No. 10207748035), and nitrogen values were analyzed by using an N-analyzer (rapid MAX Nex-HeAr; Elementar Analysesysteme GmbH, Langenselbold, Germany), which determines nitrogen by using the combustion method according to Dumas. The calorific value, which was determined with a calorimeter (IKA Werke GmbH & Co. KG, Staufen im Breisgau, Germany) [45], measures a temperature increase in a digestion vessel, which is equipped with a weighted sample. The specific calorific value was calculated with the following formula.

Ho = (C × DT − Qforeign1 − Qforeign2)/m

where m represents the weight of the fuel sample; C stands for the heat capacity (C-value) of the calorimeter system; DT indicates the calculated temperature increase in water in the inner vessel of the measuring cell; Qforeign1 is the correction value for the heat energy coming from the cotton thread as ignition aid; and Qforeign2 is the correction value for the heat energy from additional burning aids.

The calculation bases were regulated in accordance with German standards (DIN EN ISO 9831) [46]. Calorific values below those of the softwood study by Schmatz et al. [47] were to be expected.

### 2.5. Statistcal Analysis

The statistical analysis was performed using the Statistical Analysis System for Windows, SAS-Enterprise Guide statistical software package version 7.1 (SAS Inst., Cary, NC, USA). The BW and foot pad scores were analyzed for the individual animals; further values, i.e., litter and excreta DM contents, were analyzed and compared at the group level. For most parameters, mean values as well as the standard deviation of the mean (SD) were calculated. The interaction effect of runs was not checked in the analysis because we considered each run as a random sample of a large number of runs. A test for normal distribution was performed using the Shapiro–Wilk test, and normally distributed data were checked for significant differences with the Ryan–Einot–Gabriel–Welsch test (simple ANOVA). Not normally distributed data were checked for significant differences with a Kruskal–Wallis test followed by a Wilcoxon two-sample test. Differences with a significant level of *p* < 0.05 were considered significant.

## 3. Results

### 3.1. Growth Performance

Table 2 presents the results of BW development of broilers housed in the experimental groups and control group.

No significant differences were noted in the BW of broilers between both groups throughout the rearing period. At d 28 of life, the BW of broilers did not differ between both groups (1658 g and 1665 g for CON and EG groups, respectively). Additionally, at d 32 of life and according to slaughterhouse data (five runs), the BW did not differ significantly between both groups (2072 g vs. 2013 g for CON and EG, respectively).

Results of feed intake, water intake, water-feed ratio, mortality corrected feed conversion ratio (FCR) and the average daily gain (ADG) for the CON and EG groups are presented in Table 3.

Significant differences between both groups were noted in feed intake (92.1 g/b/d and 86.1 g/b/d for the CON and EG groups, respectively). Nevertheless, no significant differences were shown in water intake (ml/b/d) and water-feed ratio. The mortality corrected FCR differed significantly between CON and EG (1.48 and 1.40 for CON and EG, respectively). The average daily gain (g) of the broilers was not significantly different between groups (62.7 g and 61.0 g for CON and EG, respectively).

### 3.2. Litter Sampling

The results of the analysis of litter samples are presented in Table 4.

Significant differences were noted in the litter DM content between both groups at only days 7 and 14 of life. Nevertheless, no significant differences were observed in the litter DM content between both groups at days 21 and 28 of life. At d 7 of life, the nitrogen content in litter did not differ between both groups. However, at d 14 of life, the nitrogen content in the litter of the CON group showed a significantly higher value (34.2 g/kg DM) compared to that of the EG group (33.4 g/kg DM). At days 21 and 28 of life, the nitrogen content in the litter of the EG group had significantly higher values than those of the CON group. Regarding the calorific values in the litter at days 7, 14, 21 and 28 of life, these were significantly higher in the CON group in comparison to those in the EG group. Finally, the starch content in the litter of the CON group showed a significantly higher content (24.7 g/kg DM) than that of the EG group (20 g/kg DM). Nonetheless, the starch content in the litter of the EG group showed a significantly higher content (28.7 g/kg DM) than that of the CON group (22.8 g/kg DM). At d 28 of life, the starch content in the litter of the CON group had a significantly higher content (35.6 g/kg DM) than that of the EG group (32.3 g/kg DM).

The results of the analysis of DM divided into areas of comfort, drinkers, and feeders are presented in Table 5.

Significant differences in the area of comfort between CON and EG were noted on days 7 and 14 of life. Therefore, no significant differences in the comfort area in DM were observed between both groups at days 21 and 28 of life. In the area of drinkers, significant differences in DM between both groups were noted on days 7, 14, 21, and 28 of life. On day 7 of life, the DM in the area of feeders in CON showed a significantly lower value (834 g/kg) compared to that in EG (867 g/kg). However, on days 14 and 21 of life, no significant differences in DM between CON and EG in the area of feeders were observed. Finally, on day 28, significant differences in DM were noted with a lower value in CON (774 g/kg) compared to EG (846 g/kg) in the area of feeders.

The results of nitrogen analysis between both groups, divided into areas of comfort, drinkers and feeders, are presented in Table 6.

The nitrogen analysis in the comfort area differed significantly, with higher values in CON compared to EG at days 7 and 14 of life. On day 21 of life, a significant difference between both groups with a lower nitrogen value in CON (37.1 g/kg DM) compared to EG (37.9 g/kg DM) in the area of comfort was observed. No significant difference in the nitrogen analysis between both groups in the area of comfort was noted on day 28 of life. Regarding the nitrogen analysis in the area of drinkers, no significant difference was observed on day 7 of life. Therefore, on days 14 and 21 of life, significant differences with higher nitrogen values in CON compared to EG were noted. On day 28 of life, the measured nitrogen in the area of drinkers was significantly lower in CON (43.0 g/kg DM) compared to the value of EG (44.9 g/kg DM). Finally, in the area of feeders, no significant differences in the nitrogen value between both groups were noted on days 14 and 28 of life. However, significant differences at day 7 of life with higher nitrogen values in CON (27.7 g/kg DM) compared to EG (22.7 g/kg DM) in the area of feeders were observed. Additionally, at day 21 of life, significant differences between CON and EG with a lower nitrogen value in CON (36.0 g/kg DM) compared to EG (37.1 g/kg DM) in the area of feeders were measured.

The results of the analysis of the calorific values between both groups, divided into areas of comfort, drinkers, and feeders, are presented in Table 7.

Significant differences in the comfort area between CON (17,356 J/g DM) with a higher calorific value compared to EG (17,039 J/g DM) were observed on day 28 of life. Nevertheless, no significant differences between both groups in the area of comfort were noted on days 7, 14, and 21 of life. On days 7, 14, 21, and 28 of life, the area of drinkers measured significant differences, with higher calorific values in CON compared to EG.

### 3.3. Footpad Health

The FPD scores for broilers throughout the rearing period are presented in Table 8.

On d 7 and d 14 of life, birds housed in CON group showed significantly lower FPD scores compared to those reared in EG group (0.73 vs. 1.47 at d 7; 1.46 vs. 2.31 at d 14). However, at days 21 and 28 of life, birds housed in the experimental barn (EG) had significantly lower FPD scores in comparison to those in the CON group (2.11 vs. 2.88 on d 28 of life). Similarly, on d 32 of life and according to slaughterhouse data (five runs), the FPD scores for broilers housed in the CON group showed significantly higher scores (22.2) than those housed in the EG group (8.80).

## 4. Discussion

In this study, installing slatted flooring placed underneath the waterlines in littered broiler barns was evaluated concerning broiler growth performance, litter quality, and foot pad health.

### 4.1. Impact on Growth Performance

No significant differences in body weight were found between control and experimental groups during the fattening period. According to Aviagen’s guidelines for the BW of Ross 308, an average weight of 2041 g can be expected for a fattening period lasting 33 days [40]. Therefore, the findings of our study are in line with Li et al. [33], who demonstrated that slatted flooring shows no loss in growth performance. In contrast to the previous studies of Almeida et al. [34], Çavusoglu et al. [35], and Chuppava et al. [28], the final BW was not improved in the current study. A reason for a non-significant weight change in the experimental group could be the number of slatted floors since in the present study, there was no complete perforated floor in any of the barns. In contrast to Chuppava et al. [28], where partially (2655 g) and fully slatted flooring (2698 g) had a positive effect on BW compared to a littered floor (2555 g), this outcome could not be observed in this study. A potential explanation is that in the current study, slatted flooring was only placed underneath waterlines, and the expanse of slatted flooring was, therefore, more limited. Nevertheless, no negative effect on BW could be detected. Further studies are needed to validate the possible reasons for differences in weight gain during the entire fattening period. Regarding two more performance parameters besides weight gain, specifically feed intake and the mortality corrected FCR, significant differences were found between both groups. Both parameters were significantly higher in CON compared to EG. In general, various factors, such as ingredients, dietary supplements, exogenous feed enzymes, breed, age, and housing conditions [48,49,50], influence growth performance. Coates et al. [51] also found differences in performance under the same rearing conditions in feed conversion. Regardless of feed intake, Chuppava et al. [28] mentioned no significant differences in the FCR or in the water:feed ratio between partially slatted and fully slatted flooring compared to a littered floor. Blokhuis et al. [52] state that slatted flooring might offer decreased possibilities for the birds to peck on the ground, resulting in higher food pecking and feed intake. In opposition to that, the findings of this study do not support this thesis. One explanation for the observations above could be that between the slatted flooring in this project, there was enough space in the area of comfort and feeders for food pecking.

### 4.2. Impact on Litter Quality

Litter quality was assessed in this study by analyzing the samples for dry matter, nitrogen, calorific value, and starch.

From the dry matter results (see Table 4 and Table 5), it can be concluded that the slatted flooring beneath the watering line can interrupt the process of the litter getting wet to a certain extent. This initially slows down the deterioration of the litter quality since the DM values on day 7 and day 14 were significantly higher in the experimental group (day 7: 760.19 g/kg; day 14: 689.62 g/kg) than in the control group (day 7: 703.32 g/kg; day 14 635.95 g/kg). If the barns are divided into areas, the same picture is reflected in the area of comfort within the first two weeks, with drier litter in the EG. In addition, the area of drinkers on all sampling days showed a significantly higher DM, especially at the end of the fattening period in the EG, with 495 g/kg compared to CON with 422 g/kg on day 28 of life. Therefore, the DM samples in the experimental barn below the drinkers indicate a positive effect, with higher DM values because, as suspected, the slatted flooring catches the water losses in the litter [53] and excrement below, and thus, the animals have less contact. In this case, the excrements lie loosely under the slatted flooring so that they are ventilated, and the evaporation is high. Thus, a possible drying takes place. As a result, values in the DM in the water area are significantly higher in the experimental group than in the control group on all days (see Table 5). The dried excrements accumulate beneath the slatted flooring to a large extent. Therefore, at the end of the fattening period, the slatted flooring can be easily lifted to the ceiling by winches, and the litter can be removed unproblematically.

Excrements have a strong influence on litter moisture and quality. The diet of the animals has a major impact on their excreta consistency and, thus, indirectly on litter parameters [7]. According to Garcês et al. [53], other influences of moist litter can be condensation, leakage in the drinker line, and air absorption. In this regard, the raised slatted flooring beneath the drinker line not only provides an area that animals prefer [54], but prevents leaks in the drinker line from entering the litter. According to Garcês et al. [53], an ideal litter would be one that absorbs liquids such as water and fecal water and dries quickly or releases the liquid. Furthermore, Pereira et al. [55] observed that there is air circulation between litter layers, which reduces the heat stress of broilers.

Nitrogen levels, on the other hand, provide information about the proportion of excrement in the litter. Nitrogen values of excreta samples, with the least possible amount of visual contamination, increased in the control group from 34.5 g/kg DM on day 7 to 44.4 g/kg DM on day 28, with similar values being seen in the experimental group with 36.9 g/kg DM on day 7 and 41.5 g/kg DM on day 28. Accordingly, the litter values of nitrogen content reflected a similar trend (see Table 4). The nitrogen concentrations increased in the course of the fattening period at the same time as the feed change, only at the beginning with a significantly lower nitrogen concentration since the litter still contained a low amount of excrement [56]. Total nitrogen values between 23.8 to 47.0 g/kg DM were shown in the study by Qafoku et al. [57]. The division into areas (drinkers, feeders and comfort) showed the same increase in each area. It is impossible to draw a final conclusion concerning the hypothesis that the amount of excreta is less in areas of comfort in the EG compared to CON, since only the first two weeks show this trend instead of the entire fattening period. Furthermore, the fact that the amount of excreta in areas of drinkers is higher in CON compared to the EG was only noted on days 14 and 21 of life. For a better understanding of these significant differences (see Table 6), further studies are needed.

With respect to calorimetry, the calorific value samples of wood shavings, which had no contact with broilers, showed average values of 20,570 J/g DM. In contrast, excreta samples that had the least possible amount of visual contamination showed average values of 16,988 ± 362 J/g DM in CON and 16,674 ± 396 J/g DM in the EG. The calorific values of the litter from the start at day 7 with values of 18,137 J/g DM in the control and 17,974 J/g DM in the experimental group decreased in the course of the fattening period until day 21. Afterwards, a small increase in calorific values on day 28 compared to day 21 in both groups has to be mentioned, with 17,421 J/g DM in CON compared to 16,975 J/g DM in the EG. Therefore, the litter values are between the calorific values of wood shavings and excreta samples. The slatted flooring had a negative impact on the calorific value on all days in barn comparison. The same negative impact with lower values in the EG compared to CON was observed in the area of drinkers and in the comfort area on day 28. A possible explanation could be the lower calorific values of the excreta in the EG compared to CON. A similar study by Ahn et al. [58] showed values of litter-excrement mixtures between 16,830 J/g to 19,700 J/g. In order to be able to classify the values better, further studies should be carried out.

The starch values presented in Table 4 show, with the exception of day 7 in CON (24.7 g/kg DM), an increase in the course of the fattening period in both groups, with a maximum of 35.6 g/kg DM in CON compared to 32.3 g/kg DM in the EG. Due to the sampling plan, samples were almost only taken from the feeder area. Thus, differences are possibly due to picking losses or errors in feeding management.

### 4.3. Impact on Footpad Dermatitis

The footpad health between both groups showed significant differences on all scoring days (see Table 8). At an early age, the FPD score was lower for the control group than for the experimental group. One possible explanation for significant differences in the experimental group is that the footpads of the broilers have to adapt to the irregular pressure of the perforation of the slatted flooring or to the dry hard litter, causing them to show initially higher FPD scores. After that, the distribution changed so that from day 21 until the end of the fattening period, the control group had a higher score than the experimental group. A similar distribution was then observed in the camera-based footpad scoring of the slaughterhouse, with footpad scores in the control group of 22.20 and in the experimental group of 8.80. In general, footpad health serves as an indicator of housing conditions and animal welfare of broilers [4] and is dependent on the moisture of the litter in the broiler house, according to Mayne et al. [12]. Martland et al. [29] and Ekstrand and Algers [30] also describe similar typical studies where the footpad value continuously increases due to the increased moisture in the litter. Da Costa et al. [32] described other influences, such as mechanical pressure on the footpads. Similar results with perforated levels, with the broilers showing a lower footpad dermatitis frequency, are seen in the study by Çavusoglu et al. [35] for fully slatted flooring with 0% footpad dermatitis. These aforementioned results contrast with the negative footpad results of Almeida et al. [59] or the study of Chuppava et al. [28], which showed no effects on footpad health, with FPD scores of 0.40 on litter, 0.64 on partially slatted flooring, and 0.59 on fully slatted flooring. In conclusion, the control group, as expected, showed an increase in FPD score towards the end of the fattening period in opposition to the experimental group, with higher scores only in the early days. Da Costa et al. [32] demonstrated an adaptation of the footpads to perforated flooring due to its increased mechanical pressure. Accordingly, the significant differences described can result from the fact that the footpads of the broilers have to adapt to the irregular pressure of the perforation of the slatted flooring or to the dry hard litter in the early fatting period, and they therefore initially show a higher FPD score. In the course of fattening, however, these areas of the footpad heal and due to the drier litter in the rest of the barn, the typical continuous deterioration of the footpads occurs later [29,30]. This result of improved footpad health of the experimental group was also reflected in the camera-based footpad scores of the slaughterhouse data.

## 5. Conclusions

This study showed that slatted flooring beneath the waterline has no significant negative influence on the BW of broilers but significant positive effects on footpad health. The condition of the litter, in terms of infiltration and its quality, was mainly reflected by the DM values. Therefore, the moisture of the litter is in most parts of the barn significantly drier, especially in the drinker area. In this study, a perforated area beneath the watering lines shows a positive effect on litter quality, especially in the first two weeks of fattening, and a positive effect on footpad health from day 21 to the slaughter age of broilers and should be verified and established in future experimental runs. Further studies should exclude study limitations of this publication, such as including a slower-growing strain with a lower stocking density and a longer fattening period.

## Figures and Tables

**Table 1:** Chemical composition of the commercial diets in accordance with the ingredient declaration.

Item (%)	Starter	Rearing I	Rearing II	Finisher
Crude protein	21.60	19.00	19.00	19.50
Crude fat	5.80	4.50	4.70	8.20
Crude fiber	2.50	3.50	3.20	3.30
Crude ash	5.50	5.20	5.10	4.70
Calcium	0.90	0.75	0.70	0.60
Phosphorus	0.65	0.55	0.50	0.40
Sodium	0.16	0.16	0.15	0.14
Lysine	1.36	1.12	1.12	1.14
Methionine	0.60	0.52	0.54	0.29
Methionine hydroxy-analog	0.00	0.00	0.00	0.23
^1^ Metabolizable energy (MJ/kg)	12.60	12.20	12.60	13.20

^1^ Metabolizable energy (MJ/kg) = 0.01551 × g/kg crude protein + 0.03431 × g/kg crude fat + 0.01669 × g/kg starch + 0.01301 × g/kg sugar [41].

**Table 2:** Development of body weight (g) of broilers (*n* = 5) from d 1 to d 28 of life on farm and the final BW from slaughterhouse data (*n* = 5 runs) (mean ± SD).

Day of Life	Groups		*p*-Value
	CON	EG	
1	51.1 ^a^ ± 2.56	49.1 ^a^ ± 4.25	0.4025
7	198 ^a^ ± 19.4	197 ^a^ ± 8.90	0.8825
14	516 ^a^ ± 44.0	514 ^a^ ± 19.5	0.9224
21	1030 ^a^ ± 65.7	1006 ^a^ ± 91.8	0.6496
28	1658 ^a^ ± 78.9	1665 ^a^ ± 87.5	0.9011
32 (slaughterhouse data)	2072 ^a^ ± 88.2	2013 ^a^ ± 71.1	0.2750

CON = control group; EG = experimental group with slatted flooring beneath the waterline. ^a^ = Means within the same row with different superscripts differ significantly (*p* < 0.05).

**Table 3:** Feed intake (g/bird/day), water intake (ml/bird/day), water/feed ratio, mortality corrected FCR, and average daily gain (g) of broilers from five runs (mean ± SD).

Item	Groups		*p*-Value
	CON	EG	
Feed intake (g/b/d)	92.1 ^a^ ± 2.85	86.1 ^b^ ± 3.99	0.0324
Water intake (ml/b/d)	206.1 ^a^ ± 4.15	197.1 ^a^ ± 18.28	0.3176
Water-feed ratio	2.24 ^a^ ± 0.09	2.25 ^a^ ± 0.14	0.8824
FCR	1.48 ^a^ ± 0.02	1.40 ^b^ ± 0.03	0.0078
ADG (g)	62.7 ^a^ ± 2.65	61.0 ^a^ ± 2.22	0.3237

CON = control group; EG = experimental group with slatted flooring beneath the waterline. ^a, b^ = means within the same row with different superscripts differ significantly (*p* < 0.05).

**Table 4:** Dry matter (DM) (g/kg), nitrogen (g/kg), calorific value (J/g) and starch (g/kg) of litter from d 7 to d 28. Nitrogen, calorific value and starch are expressed on dry matter basis (mean ± SD).

Item	Day of Life (*n* = CON/EG)	Groups		*p*-Value
		CON	EG	
DM (g/kg)	7 (*n* = 255/235)	703 ^b^ ± 169	760 ^a^ ± 165	0.0002
	14 (*n* = 255/235)	636 ^b^ ± 198	690 ^a^ ± 167	0.0013
	21 (*n* = 255/235)	640 ^a^ ± 215	627 ^a^ ± 182	0.4583
	28 (*n* = 255/235)	604 ^a^ ± 207	594 ^a^ ± 183	0.5676
Nitrogen (g/kg DM)	7 (*n* = 255/235)	27.7 ^a^ ± 6.08	26.6 ^a^ ± 7.67	0.0736
	14 (*n* = 255/235)	34.2 ^a^ ± 2.84	33.4 ^b^ ± 2.71	0.0018
	21 (*n* = 255/235)	38.0 ^b^ ± 2.56	38.7 ^a^ ± 3.56	0.0159
	28 (*n* = 255/235)	41.8.0 ^b^ ± 2.56	43.8 ^a^ ± 3.04	0.0001
Calorific value (J/g DM)	7 (*n* = 195)	18137 ^a^ ± 732	17974 ^b^ ± 771	0.0334
	14 (*n* = 195)	17352 ^a^ ± 321	17251 ^b^ ± 309	0.0018
	21 (*n* = 195)	17122 ^a^ ± 321	16950 ^b^ ± 340	0.0001
	28 (*n* = 195)	17421 ^a^ ± 415	16975 ^b^ ± 333	0.0001
Starch (g/kg DM)	7 (*n* = 95)	24.7 ^a^ ± 17.2	20.0 ^b^ ± 13.9	0.0437
	14 (*n* = 95)	20.7 ^a^ ± 7.25	21.6 ^a^ ± 12.6	0.5537
	21 (*n* = 95)	22.8 ^b^ ± 4.05	28.7 ^a^ ± 13.6	0.0001
	28 (*n* = 95)	35.6 ^a^ ± 10.4	32.3 ^b^ ± 9.43	0.0254

CON = control group; EG = experimental group with slatted flooring beneath the waterline. ^a, b^ = Means within the same row with different superscripts differ significantly (*p* < 0.05).

**Table 5:** Dry matter (g/kg) of litter in comfort, drinker, and feeder areas from d 7 to d 28 (mean ± SD).

Area	Day of Life (*n* = CON/EG)	Groups		*p*-Value
		CON	EG	
Comfort	7 (*n* = 100/60)	779 ^b^ ± 49.7	833 ^a^ ± 65.8	0.0001
	14 (*n* = 100/60)	722 ^b^ ± 76.6	772 ^a^ ± 61.4	0.0001
	21 (*n* = 100/60)	749 ^a^ ± 77.6	729 ^a^ ± 87.0	0.1351
	28 (*n* = 100/60)	693 ^a^ ± 133	714 ^a^ ± 86.7	0.2795
Drinkers	7 (*n* = 80/120)	606 ^b^ ± 96.9	756 ^a^ ± 85.1	0.0001
	14 (*n* = 80/120)	450 ^b^ ± 83.8	650 ^a^ ± 95.8	0.0001
	21 (*n* = 80/120)	416 ^b^ ± 51.9	548 ^a^ ± 106	0.0001
	28 (*n* = 80/120)	422 ^b^ ± 78.9	495 ^a^ ± 79.0	0.0001
Feeders	7 (*n* = 60/40)	834 ^b^ ± 39.2	867 ^a^ ± 37.9	0.0001
	14 (*n* = 60/40)	844 ^a^ ± 39.4	858 ^a^ ± 75.8	0.2217
	21 (*n* = 60/40)	864 ^a^ ± 26.6	860 ^a^ ± 30.0	0.5313
	28 (*n* = 60/40)	774 ^b^ ± 160	846 ^a^ ± 35.1	0.0069

CON = control group; EG = experimental group with slatted flooring beneath the waterline. ^a, b^ = Means within the same row with different superscripts differ significantly (*p* < 0.05). Comfort = area with dry litter where the broilers can pick, scratch, and take a dust bath.

**Table 6:** Nitrogen (g/kg DM) of the litter in comfort, drinker, and feeder areas from d 7 to d 28 (mean ± SD).

Area	Day of Life (*n* = CON/EG)	Groups		*p*-Value
		CON	EG	
Comfort	7 (*n* = 100/60)	24.5^a^ ± 6.39	21.2 ^b^ ± 6.83	0.0023
	14 (*n* = 100/60)	33.9 ^a^ ± 2.30	32.5 ^b^ ± 2.15	0.0001
	21 (*n* = 100/60)	37.1 ^b^ ± 1.54	37.9 ^a^ ± 1.60	0.0036
	28 (*n* = 100/60)	41.1 ^a^ ± 2.73	41.5 ^a^ ± 2.43	0.3764
Drinkers	7 (*n* = 80/120)	30.5 ^a^ ± 4.97	29.3 ^a^ ± 6.29	0.1475
	14 (*n* = 80/120)	36.1 ^a^ ± 2.60	34.7 ^b^ ± 2.56	0.0003
	21 (*n* = 80/120)	40.6 ^a^ ± 1.87	40.0 ^b^ ± 2.18	0.0344
	28 (*n* = 80/120)	43.0 ^b^ ± 1.89	44.9 ^a^ ± 2.42	0.0001
Feeders	7 (*n* = 60/40)	27.7 ^a^ ± 4.03	22.7 ^b^ ± 6.04	0.0001
	14 (*n* = 60/40)	32.0 ^a^ ± 2.19	31.7 ^a^ ± 2.37	0.4673
	21 (*n* = 60/40)	36.0 ^b^ ± 1.34	37.1 ^a^ ± 1.47	0.0001
	28 (*n* = 60/40)	40.9 ^a^ ± 1.82	40.9 ^a^ ± 1.39	0.8863

CON = control group; EG = experimental group with slatted flooring beneath the waterline. ^a, b^ = Means within the same row with different superscripts differ significantly (*p* < 0.05). Comfort = area with dry litter where the broilers can pick, scratch, and take a dust bath.

**Table 7:** Calorific values (J/g DM) of the litter in comfort and drinker areas from d 7 to d 28 (mean ± SD).

Area	Day of Life (*n* = CON/EG)	Groups		*p*-Value
		CON	EG	
Comfort	7 (*n* = 100/60)	18282 ^a^ ± 610	18427 ^a^ ± 880	0.2238
	14 (*n* = 100/60)	17365 ^a^ ± 293	17308 ^a^ ± 262	0.2228
	21 (*n* = 100/60)	17052 ^a^ ± 244	17019 ^a^ ± 288	0.4330
	28 (*n* = 100/60)	17356 ^a^ ± 386	17039 ^b^ ± 305	0.0001
Drinkers	7 (*n* = 80/120)	18206 ^a^ ± 682	17879 ^b^ ± 537	0.0002
	14 (*n* = 80/120)	17410 ^a^ ± 299	17281 ^b^ ± 287	0.0026
	21 (*n* = 80/120)	17268 ^a^ ± 339	16963 ^b^ ± 340	0.0001
	28 (*n* = 80/120)	17542 ^a^ ± 404	16998 ^b^ ± 304	0.0001

CON = control group; EG = experimental group with slatted flooring beneath the waterline. ^a, b^ = Means within the same row with different superscripts differ significantly (*p* < 0.05). Comfort = area with dry litter where the broilers can pick, scratch, and take a dust bath.

**Table 8:** Footpad dermatitis score of broilers (*n* = 250) from d 7 to d 28 (mean ± SD).

Day of Life	Groups		*p*-Value
	CON	EG	
7	0.73 ^b^ ± 0.84	1.47 ^a^ ± 1.17	0.0001
14	1.46 ^b^ ± 1.23	2.31 ^a^ ± 1.32	0.0001
21	2.56 ^a^ ± 1.68	1.91 ^b^ ± 1.48	0.0001
28	2.88 ^a^ ± 1.82	2.11 ^b^ ± 1.51	0.0001

CON = control group; EG = experimental group with slatted flooring beneath the waterline. ^a, b^ = Means within the same row with different superscripts differ significantly (*p* < 0.05).

## Data Availability

The original contributions generated for the study are included in the article; further inquiries can be directed to the corresponding author.
